# Prevalence of malaria-helminth co-infections among children living in a setting of high coverage of standard interventions for malaria and helminths: Two population-based studies in Senegal

**DOI:** 10.3389/fpubh.2023.1087044

**Published:** 2023-03-02

**Authors:** Muhammed O. Afolabi, Doudou Sow, Ibrahima Mbaye, Marie Pierre Diouf, Mor Absa Loum, Elhadji Babacar Fall, Amadou Seck, Isaac A. Manga, Cheikh Cissé, Baba Camara, Awa Diouf, Ndéye Aida Gaye, Aminata Colle Lo, Brian Greenwood, Jean Louis A. Ndiaye

**Affiliations:** ^1^Department of Disease Control, London School of Hygiene and Tropical Medicine, London, United Kingdom; ^2^Service de Parasitologie-Mycologie, Université Gaston Berger de Saint-Louis, Saint-Louis, Senegal; ^3^Service de Parasitologie-Mycologie, Université de Thies, Thies, Senegal; ^4^Service de Parasitologie-Mycologie, Université Cheikh Anta Diop, Dakar, Senegal; ^5^Saraya Health Centre, Saraya, Senegal

**Keywords:** burden, co-endemicity, geohelminths, malaria, bilharzia (schistosomiasis), Sub-Saharan Africa

## Abstract

**Background:**

Concurrent infections of *Plasmodium falciparum* with Soil Transmitted Helminths (STH) and *Schistosoma spp* are still a major public health problem among children living in Sub-Saharan Africa. We conducted two prospective studies among children living in urban and rural settings of Senegal, where control programmes for malaria, STH and schistosomiasis have been sustained, to determine the prevalence of malaria-helminth co-infection.

**Methods:**

We enrolled 910 children aged 1–14 years from Saraya and Diourbel districts of Senegal in June and November 2021, respectively. We collected finger-prick blood samples from the children for malaria parasite detection using microscopy and PCR methods. Stool samples were also collected and Kato-Katz and PCR methods were used to detect STH and *S. mansoni;* and Merthiolate-iodine-formalin (MIF) test for other intestinal protozoans. Urine samples were analyzed using a filtration test, Point of Care Circulating Cathodic Antigens (POC-CCA) and PCR methods for detection of *S. haematobium*. Statistical analyses were performed to compare the continuous and categorical variables across the two study sites and age groups, as well as using the adjusted Odds ratios (aOR) to explore risk factors for malaria-helminth co-infections.

**Results:**

The overall prevalence of polyparasitism with *P. falciparum*, STH, *S. haematobium* and *S. mansoni* among children in the two study sites was 2.2% (20/910) while prevalence of *P. falciparum-S. haematobium* co-infection was 1.1% (10/910); *P. falciparum-S. mansoni* 0.7% (6/910) and *P. falciparum* with any intestinal protozoan 2.4% (22/910). Co-infection was slightly higher among 5–14 year old children (17/629, 2.7%; 95% CI: 1.43–3.97) than 1–4 years (3/281, 1.1%; 95% CI: −0.12–2.32) and, in boys (13/567, 2.3%; 95% CI: 1.27–3.96) than girls (7/343, 2.1%; 95% CI: 0.52–3.48). Children aged 5–14 years (aOR = 3.37; 95% CI: 0.82–13.77, *p* = 0.09), who were boys (aOR = 1.44; 95% CI: 0.48–4.36, *p* = 0.51) and lived in Saraya (aOR = 1.27; 95% CI: 0.24–6.69, *p* = 0.77) had a higher risk of malaria-helminth co-infection than other age group, in girls and those who lived in Diourbel. Living in houses with spaces between the walls and roofs as well as frequent contacts with water during swimming were statistically significant risk factors for malaria-helminth co-infection.

**Conclusions:**

The prevalence of malaria-helminth co-infection is low in two districts in Senegal, possibly due to sustained implementation of effective control measures for malaria and NTDs. These findings could help to develop and implement strategies that would lead to elimination of malaria and helminths in the study areas.

## Introduction

Co-existence of two or more parasitic infections in a human host has been described in the literature as “polyparasitism” or “multiparasitism” (1, 2). This concept of concomitant infections with multiple species of parasites is well documented in children living in low-and middle-income countries (LMIC) (3, 4). Apart from the high burden of single parasitic infections and their associated health consequences, concurrent infections with *Plasmodium falciparum*, soil transmitted helminths (STH) and/or *Schistosomes* impact child growth and development (5, 6). Climatic factors and poor socio-economic conditions that support the persistence of the malaria parasite vectors and the infective larval stages of STHs and *Schistosoma spp* have been widely reported to favor the geographic overlap of these multiple parasitic infections, especially in Sub-Saharan Africa (SSA) (4). Despite the devastating impact of both malaria and helminth infections on child survival, vertical control programmes targeting single parasitic infection are still being implemented in most co-endemic settings in SSA. Historical data have shown that coverage of these control programmes fluctuates and their effectiveness varies depending on the preventive chemotherapy used and the duration between cycles (7). In addition, variations in uptake of chemotherapy have contributed to the survival of parasites in certain areas and age groups, mainly among children (8).

Although a reduction in the prevalence of mono-infection with *Plasmodium spp* (9, 10), and STH (11, 12) has been reported in many African communities as a result of sustained implementation of various control strategies, concurrent infections with malaria, STH and schistosomiasis still remain a significant public health threat. In such co-endemic settings, vertical control measures that target a single parasitic infection may not be the optimum control strategy to interrupt the transmission cycle of the parasites.

Monitoring the prevalence of parasitic infections is recommended before and after the implementation of a control campaign in order to understand the transmission dynamics of the targeted parasite at the population level. However, in many countries with co-endemic parasitic infections, control programmes are often implemented without comprehensive baseline data, mainly because of financial challenges and logistical factors related to undocumented channels of accessing the control medications (13). In addition, most impact assessment studies deploy traditional diagnostic methods which tend to underestimate the prevalence of parasitic infections, especially in low transmission settings. Given that accurate diagnostic tools play a pivotal role in monitoring of treatment efficacies in mass drug administration (MDA) programmes, there is a need for the deployment of improved, yet simple and cost-effective diagnostic tool to detect mixed infections in a single reaction (14). This approach will provide reliable estimates of the prevalence of co-existing parasitic infections, in line with the goals of the new WHO 2030 NTD road map which now focuses on eliminating STH and schistosomiasis as a public health problem (11). The WHO Global technical strategy for malaria 2016–2030, which sets the target of reducing global malaria incidence and mortality rates by at least 90% by 2030 and accelerate progress toward malaria elimination is consistent with the 2030 NTD road map (15).

Given the changing landscape of transmission of malaria, STH and schistosomiasis in SSA, integrated approaches to tackle multiply-related infectious diseases are key WHO recommendations to achieve the 2030 targets. In 2022, the Kigali Declaration pledged USD 4.25 billion to support an end to malaria and NTDs by 2030 (16). Given this development, obtaining reliable data on the prevalence of co-infection with malaria, STH and schistosomiasis across endemic areas becomes an increasingly important prerequisite to developing locally appropriate control strategies that could interrupt transmission of these infections. We designed the studies reported in this paper to measure the co-prevalence of helminths and malaria in two areas of Senegal as a prelude to the design of an integrated control strategy.

## Methods

### Study design, settings and participants

**Figure 1:**
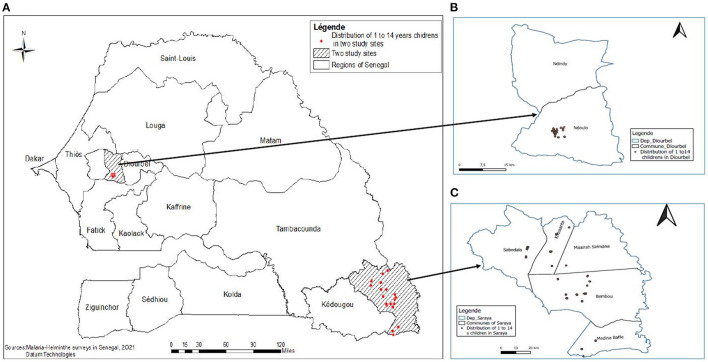
**(A–C)** Map of Senegal showing the two study sites in Saraya and Diuorbel districts.

We conducted two prospective, population-based studies among pre-school and school-aged children in Saraya and Diourbel districts of Senegal in June and November 2021, respectively. The study period did not correspond to a peak of malaria transmission in the two study sites. Further details about the settings and population for these study areas have been described elsewhere (17). Briefly, Saraya is a rural settlement in the south-eastern region of Senegal while Diourbel, in the western region of the country is largely sub-urban. Saraya and Diourbel are about 740 km south and 134 km east of Dakar, the capital of Senegal, respectively (Figure 1). The two communities share similar epidemiological profiles. Saraya and Diourbel districts have a tropical Sudano-Sahelian climate with well-defined dry and humid seasons that result from northeast winter winds and southwest summer winds. There are two seasons: a dry season from November to May and a rainy season from May to November in Saraya and in Diourbel, rains starts in July. Mean daily temperatures range from 14 to 36°C from July to February and 21–40°C from March to June. While malaria, STH and schistosomiasis have almost been eliminated in most districts in Senegal, Diourbel and Saraya were parts of communities that had a persistent, high burden of malaria (18) and helminths (19). In both districts, control programmes for STH and schistosomiasis have been implemented annually since 2014 (20). Also, Seasonal Malaria Chemoprevention (SMC) was introduced in 2013 in Saraya district and from 2019 in Diourbel district, with high levels of coverage achieved in both districts (20).

This study was conducted among pre-school and school-aged children (1–14 years) of either gender, who had been resident in the study areas for at least 6 months and whose parents or primary caregivers consented to their participation in the study. In Saraya site, the primary caregivers were the parents and guardians of these children. In Diourbel site, pre-school and school aged children lived as full boarders in Koranic schools (also called “dahras”) where they received Arabic education. The primary caregivers of the children in these schools were Koranic teachers, heads of Koranic schools and their wives.

### Sampling and sample size estimation

**Figure 2:**
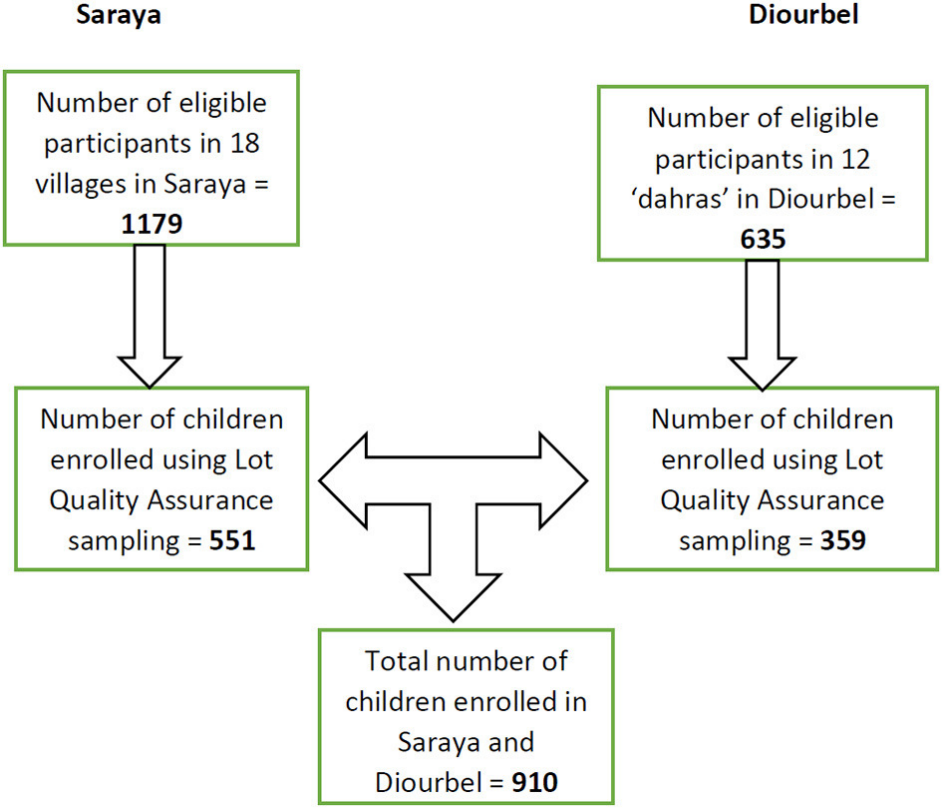
Flow chart showing enrolment process of study participants in Saraya and Diourbel, 2021.

Given that the prevalence of malaria-helminth co-infections was reported to be 13–25% among West African children (21–24), a minimum total sample size of 500 male and female children was needed to show that the variation of true prevalence was not more than 5% when improved diagnostic tools were used to detect the co-infection. Using multi-stage sampling, we selected representative households in Saraya district and Koranic schools in Diourbel district. We subsequently collected household information and the number of male and female children by age group (1–4 and 5–14 years) in the selected households and schools in the two sites. Next, we selected randomly the households or schools in the enumeration databases. To ensure a fair representation of households in the study sites, the Lot Quality Assurance Sampling technique (25) was used to recruit study participants. For example, a total of 15 children, with a roughly even mix of boys and girls, ranging in ages from 1–4 and 5–14 years, was randomly selected in each household within the selected village or Koranic school (Figure 2).

### Implementation of the studies

The studies were conducted in collaboration with the SMC and NTD programmes of the Senegal Ministry of Health and Social Action, which provided strategic support that facilitated community acceptance and mobilization. Prior to the commencement of the studies, engagement meetings with the community leaders, heads of households and parents/care-givers of potentially eligible children were undertaken to explain the study, using a simple picture depicting the burden and impact of concomitant infections with malaria and helminth in children, its rationale and the informed consent procedure, including risk and benefits of allowing children to participate in the study. After each meeting, trained research assistants identified parents/caregivers of potential study participants to explain the study further to them on an individual basis. Parents/caregivers who considered that the study was appropriate for their child/ward were visited at home at a mutually agreed time for further engagements and a formal consent procedure was completed. After obtaining written informed consent from a parent/primary care-giver, the research staff who were native speakers of a local language spoken widely in the community, administered a purpose-designed electronic questionnaire to the parents/care-givers using the local language of preference. The questionnaire covered information on socio-demographic features, health and residence characteristics, household wealth, bed net use, history of deworming and malaria treatment, environmental and behavioral factors predisposing children to co-infections with malaria and helminths. Finger-prick blood samples were collected from each participant for thick and thin smear microscopy, and filter paper blots were collected for *Plasmodium* DNA isolation and PCR amplification for species determination. Also, freshly voided urine and stool samples were collected for microscopic detection of *Schistosoma* spp and STH eggs.

### Clinical evaluation

To comply with COVID-19 public health measures that were in place at the time of the studies, body temperature was measured using a digital, non-contact, infra-red thermometer. A study participant was considered febrile, if the temperature was ≥ 37.5 °C. Height was measured to the nearest 0.1 cm using a wall-mounted stadiometer; weight was measured to the nearest 0.5 kg using a digital weighing scale, and mid-upper arm circumference (MUAC) was measured using a graduated, color-coded tape recommended by UNICEF. These measurements were used to estimate anthropometric indices including weight-for-age; height for-age and weight-for height. Anthropometric indices were computed as z-scores based on the WHO growth reference curves using the WHO AnthroPlus 1.0.4 for personal computers manual. Underweight was defined as a weight-for-age (WA) z score of < – 2, wasting as a weight-for-height (WH) z score of < – 2 and stunting as height-for-age (HA) z score of < – 2. A child was categorized as being malnourished, if they scored < – 2 in one of the anthropometric indices of WA, HA and WH indices (26).

### Malaria parasite diagnosis

Blood was collected by finger-prick for malaria parasite detection. Thick and thin blood films were prepared following standard operational procedures. Thin blood films were fixed in methanol and thick blood films were de-hemoglobinised in water before Giemsa staining. The blood films were examined microscopically following standard procedures (27). Slides were considered positive when asexual forms and/or gametocytes of any *Plasmodium* species were observed on the blood film. Two experienced microscopists read all slides independently. Malaria parasite density per μl of blood was determined by counting the number of parasites per 200 leukocytes and multiplying by an average value of white blood cell count, considered to be 8,000/ul. Parasitaemia was classified as low (≤ 500 parasite/μl of blood), moderate (501–5000 parasites/μl of blood) and high (> 5000 parasites/μl of blood).

### Urine analysis for schistosome eggs

A freshly voided urine sample was collected from each study participant into a pre-labeled plastic container with a screw cap. The urine sample was drawn from the plastic container using a syringe and filtered through a polycarbonate membrane filter (STERLITECH Corporation, Washington, USA). The filter membrane was examined microscopically for the presence of schistosome eggs (27). Ten percent of negative samples and all positive samples were re-read by a senior laboratory staff member immediately after the first reading to validate the results. Schistosome egg density was expressed as the number of eggs in 10 ml urine (eggs/10 ml) and the intensity of infection was categorized as either light (< 50 eggs/10 ml) or heavy infection (≥ 50 eggs/10 ml) (28).

In addition, parallel testing for schistosome circulating cathodic antigens (CCA) in urine was undertaken (29). The urine CCA dipstick test has been extensively used in SSA and has sensitivity and specificity values ranging from 52.5 to 63.2% and 57.7 to 75.6%, respectively (30, 31). Two drops of urine were added to the circular well of the test cassette. Following 20 min of incubation, results were determined by visual reading (Schisto POC-CCA cassette based test; Rapid Medical Diagnostics, Pretoria, South Africa).

### Fecal examination by Kato-Katz method

Fresh stool samples were collected, smears were prepared, and examined using the Kato-Katz thick smear method (27). Duplicate smears were prepared for each specimen using a 41.7 mg Kato-Katz template. Each slide was allowed to clear for 30 min, and then examined at 100 × total magnification within 1 h of preparation to avoid missing hookworm eggs. The number of eggs counted per slide was multiplied by 24 to obtain the egg count per gram (epg) of feces. As a quality control measure, all positive slides and 50% of randomly selected negative smears were re-examined independently by a third parasitologist. An average of the counts was utilized. For *A. lumbricoides*, participants with 1–4 999 epgs were classified as having light infection, those with 5 000–49 999 epg as moderate, and ≥ 50 000 as heavy infections. For *T. trichuria*, participants with 1–999 epg was light infection; 1 000–9 999 epg as moderate, and ≥ 10 000 as heavy infection. The intensity of infections was classified according to WHO guidelines for *S. mansoni* infection into light (1–99 eggs/g of stool (epg), moderate (100–399 epg) and heavy infections (≥400 epg) (28).

### MIF technique for detection of intestinal protozoa

The traditional fixation method, MIF technique (32), was used to further examine the stool samples. The 2 × 24 Copro-Duo Kit (RAL Diagnostics, France) was used to highlight protozoan cysts, schistosome eggs and unfertilized *Ascaris* eggs. Stool samples were added to cryotubes to which Mercurothiolate, Iodine and Formalin (MIF) solution (4 drops of Lugol in 15 microliters of MIF) was added. The samples were stored at +4°C, smears prepared and examined for parasite eggs using 10x and 40x microscope objectives.

### PCR analysis

Dried blood spot, urine and stool samples were analyzed using PCR methods described in the Supplementary file S1.

### Statistical analysis

Descriptive analyses including median and interquartile range (IQR), geometric means, frequencies, and proportions were undertaken to summarize the data. Means and proportions were constructed for continuous and categorical variables, respectively. The study participants were categorized into pre-school (1–4 years) and school aged (5–14 years) children. Co-infection was defined as infection involving *Plasmodium* spp with at least one helminth parasite. Differences in the proportions between the two study sites were compared using Pearson's Chi-squared (χ^2^) and Fisher Exact tests. Correlation coefficient (*r*) was used to establish the relationship between the different parasite densities. Uni-variable regression analyses were used to explore risk factors for malaria-helminth co-infections. Odds ratios and 95% confidence intervals were obtained for each explanatory variable and malaria-helminth co-infection in the respective logistic models. The adjusted odds ratio (aOR) in the multivariate analysis was used to establish the strength of the association of the risk factors with the co-infection. Variables with *p*-values < 0.05 were considered indicative of statistical significance. All data were analyzed using R 4.1.1 software.

### Ethical approval and consent to participate

Ethical approvals for this study were obtained from the Research Ethics Committee of the London School of Hygiene and Tropical Medicine and the Comité National d'Ethique pour la Recherche en Santé (CNERS) in Senegal. Written informed consent was obtained from parents/caregivers whose children participated in the study after explaining the purpose and benefits of their participation. Participation was voluntary, and personal data collected about the study children were anonymised, kept confidential and held in compliance with international data privacy protection laws and regulations. Participants who had positive results for *P. falciparum* were treated with a complete course of the first line treatment recommended by the Senegal national treatment guideline policy for uncomplicated malaria (artesunate-amodiaquine), participants with an STH were treated with a single dose of albendazole; and praziquantel was used to treat children who had positive results for schistosomiasis (33).

## Results

### Characteristics of study participants

From 14 to 21 June 2021, we enrolled 551 children from 18 villages of Saraya district, and 359 children in 12 randomly selected Koranic schools in Diourbel district from 3 to 6 November 2021. The median ages (IQR) for children aged 1–4 years were 3.3 (2.5–4) and 4 (3–4) in Saraya and Diourbel sites respectively. Among the 5–14 years age group, the median ages (IQR) were 8.1 (7, 8.5) and 9.3 (8, 11) in Saraya and Diourbel, respectively. An almost equal number of boys and girls were enrolled in Saraya (275/551, 49.9%) and Diourbel (276/551, 50.1%), respectively. However, 297 boys (297/359, 82.7%) and only 62 girls (62/359, 17.3%) were enrolled in Diourbel. A similar proportion of children were found to be malnourished in Saraya and Diourbel: 98/551 (17.8%) and (68/359, 18.9%), respectively. A majority of children in Saraya lived in houses owned by their parents/caregivers (506/551, 91.8%), while about three-fifths of the children (231/359, 64.3%) in Diourbel lived in the houses owned by their primary caregivers. Majority of houses where the children in Diourbel lived were made of block and cement 247/359 (68.8%) while in Saraya the majority lived in houses built from wood (233/258, 42.1%), or mud (135, 24.4%). Similar proportions of children in Diourbel and Saraya lived in houses which had no net on their doors (349/351, 97.2% vs. 542/551, 98.3%) or windows (349/359, 97.2% vs. 540/551, 98%). More than 80% of the children in Diourbel, compared to about 65% of the children in Saraya had bed nets in their houses: (302/359, 84.1% vs. 360/551, 65.3%) respectively and more than three-fifth of the children in Diourbel slept under a bed net every night (224/359, 62.4%) compared to slightly over half of the children in Saraya (312/551, 56.6%) (Table 1).

**Table 1:** Socio-demographic characteristics of study participants, Saraya and Diourbel, 2021.

**Study site**	**Diourbel (*****N =*** **359)**		**Saraya (*****N =*** **551)**	
**Characteristics**	***n*** **(%)**		***n*** **(%)**	
**Age groups**	**Median (IQR)**	***n*** **(%)**	**Median (IQR)**	***N*** **(%)**
1–4 years	4 (3–4)	10 (2.8)	3.3 (2.5–4.00)	266 (48.1)
5–14 years	9.3 (8–11)	349 (97.2)	8.1 (7.00–8.54)	285 (51.5)
**Gender**				
Male	297(82.7)		275 (49.9)	
Female	62 (17.3)		276 (50.1)	
**Nutritional status**				
Not malnourished	291 (81.1)		453 (82.2)	
Malnourished	68 (18.9)		98 (17.8)	
**Household ownership of parents/caregivers**				
Owned a house	231 (64.3)		506 (91.8)	
Rented a houses	8 (2.2)		45 (8.2)	
Shared with relatives	120 (33.4)		0 (0)	
**House walls**				
Corrugated sheet	92 (25.6)		5 (0.9)	
Mud	8 (2.2)		135 (24.4)	
Wood	2 (0.6)		233 (42.1)	
Block/cement	247 (68.8)		52 (9.4)	
Others	10 (2.8)		106 (19.2)	
Don't Know	0 (0)		22 (3.98)	
**Presence of net on the window**				
Yes	10 (2.8)		11 (2.0)	
No	349 (97.2)		540 (98.0)	
**Presence of net on the door**				
Yes	10 (2.8)		9 (1.7)	
No	349 (97.2)		542 (98.3)	
**Space between the wall and roof**				
Yes	246 (69.3)		190 (35.7)	
No	106 (29.9)		312 (58.5)	
Don't Know	3 (0.9)		31 (5.8)	
**Household wealth**				
Radio	2 (0.6)		20 (3.6)	
Television	1 (0.3)		1 (0.2)	
Mobile phone	32 (8.9)		101 (18.3)	
Fridge	1 (0.3)		0 (0)	
Radio + TV + Mobile	203 (56.5)		139 (25.1)	
Any two of the items	119 (33.2)		265 (47.9)	
None of the items	1 (0.3)		27 (4.9)	
**Means of transportation**				
Bicycle	30 (8.5)		61 (11.2)	
Motorcycle	26 (7.32)		202 (37.2)	
Motor car	0 (0)		2 (0.4)	
All the items	35 (9.9)		6 (1.1)	
Any two of the items	3 (0.84)		133 (24.5)	
None of the items	261 (73.5)		139 (25.60)	
**Availability of bed net in the households**				
Yes	302 (84.1)		360 (65.3)	
No	57 (15.9)		191 (34.7)	
**Slept under a bed net**				
Every night	224 (62.4)		312 (56.6)	
1–4 times a week	8 (2.2)		20 (3.6)	
Once a month	68 (18.9)		16 (2.9)	
Never	2(0.6)		12 (2.2)	
Don't know	39(10.9)		191(34.7)	

More than half of the children in Diourbel lived in houses where toilet facilities were shared among many family members; this proportion was higher among children in Saraya (190/359, 52.9% vs. 423/551, 76.8%). Almost all children in Diourbel and Saraya used water to clean their anus following defaecation (355/359, 98.9% and 550 /551, 99.8%), respectively. However, only about two-fifth of the children in Diourbel and one-third of Saraya children washed their hands with soap and water after defaecation (149/359, 41.5% and 157/551, 28.5%), respectively. The source of drinking water in more than 60% of children in Diourbel was from a public tap water (241/351, 67.1%) while the most common source of drinking among Saraya children was from a public borehole (237/551, 42.9%). While about 13% of children in Saraya had daily exposure to river water through activities such as swimming (76/551, 13.7%), this was reported in only two children in Diourbel (2/359, 0.6%). Playing in sandy areas was practiced by a majority of the children in both study sites (353/359, 98.3% and 488/551, 88.6%, respectively); walking barefooted was reported in 11.3% of Saraya children (61/551, 11.3%) and in six children from Diourbel (6/359, 1.7%). Slightly more than 10% of the children in Saraya reported fever in the 28 days prior to this study (72/551, 13.1%) while this was reported in only one child in Diourbel (1/359, 0.3%). Nearly half of the children in Diourbel and a quarter of the children in Saraya had received deworming drugs in the 12 months preceding the study (173/359, 48.2% and 140/551, 25.5%). Similarly, above half of Diourbel and Saraya children received antimalarial treatment more than 8 weeks before the studies (202/359, 56.4% and 391/551, 71%, respectively) (Table 2).

**Table 2:** Social and behavioral practices of study participants, Saraya and Diourbel districts, 2021.

**Study site**	**Diourbel**	**Saraya**
**Characteristics**	***n*** **(%)**	***n*** **(%)**
**Types of toilet facilities**		
Private indoor	166 (46.2)	0 (0)
Private outdoor	1 (0.3)	79 (14.3)
Shared with other families	190 (52.9)	423 (76.8)
Communal pit latrines	1 (0.3)	26 (4.7)
Others	0 (0)	4 (0.7)
No toilet facilities	1 (0.3)	19 (3.4)
**Cleaning of anus after bowel movement**		
Water	355 (98.9)	550 (99.8)
Leaves	1 (0.3)	1 (0.2)
Old newspaper	1 (0.3)	0 (0)
Tissue paper	2 (0.6)	0 (0)
**Place for washing hand after using toilet**		
Yes	136 (38.1)	219 (40.1)
No	221 (61.9, [56.63–66.92])	327 (59.9)
**Washing hands after using toilet**		
Water only	209 (58.2)	391 (70.9)
Soap and water	149 (41.5)	157 (28.5)
Others	1 (0.3)	3 (0.5)
**Frequency of washing hand after using toilet**		
When there is water	189 (64.3)	502 (97.3)
When stool is soft	4 (1.4)	14 (2.7)
Rarely	99 (33.7)	0 (0)
Never	2 (0.7)	0 (0)
**Frequency of washing hands before eating**		
Sometimes	88 (24.5)	100 (18.1)
Always	130 (36.2)	432 (78.3)
Rarely	135 (37.6)	19 (3.4)
Never	6 (1.7)	1 (0.2)
**Wash hands before last meal**		
Yes	303 (84.9)	528 (98.0)
No	54 (15.1)	11 (2.0)
**Contact with river e.g. swimming**		
Everyday	2 (0.6)	76 (13.7)
Approximately once in a week	0 (0)	209 (37.8)
Approximately once in a month	4 (1.1)	25 (4.5)
Rarely	155 (43.2)	165 (29.8)
Never	198 (55.2)	78 (14.1)
**Sources of drinking water**		
Private indoor tap	241 (67.1)	13 (2.4)
Private outdoor tap	28 (7.8)	45 (8.2)
Municipal tap water	2 (0.6)	63 (11.4)
Borehole	1 (0.3)	237 (42.9)
Well without pump	85 (23.7)	104 (18.8)
Stream or river	1 (0.3)	22 (3.9)
Others	1 (0.3)	68 (12.3)
**Walking time to standing water source**		
< 5 min	116 (32.3)	46 (8.3)
5 to 10 min	33 (9.2)	133 (24.1)
10 to 14 min	2 (0.6)	122 (22.1)
15 to 30 min	5 (1.4)	130 (23.6)
>30 min	203 (56.5)	121 (21.9)
**Shoes wearing**		
Closed shoes	11 (3.07)	20 (3.63)
Sandal	290 (81.0)	502 (91.1)
No Shoes	57 (15.9)	29 (5.3)
**Child activities**		
Play with mates in sandy area	353 (98.3)	488 (88.6)
Walk barefooted	6 (1.7)	61 (11.3)
Sleep on bare floor	0 (0.0)	2 (0.4)
**Washed hand before last meal**		
Yes	303 (84.9)	528 (98.0)
No	54 (15.1)	11 (2.0)
**History of taking deworming drugs**		
In the last 6 months	8 (2.2)	226 (41.1)
Between 6 and 12 months	12 (3.3)	155 (28.2)
In the last 12 months	173 (48.2)	141 (25.5)
Never	166 (46.2)	29 (5.3)
**Fever in the last 28 days**		
Yes	1 (0.3)	72 (13.1)
No	358 (99.7)	479 (86.9)
**History of taking antimalarial drugs**		
In the last 4 weeks	16 (4.5)	78 (14.2)
Between 4 and 8 weeks	20 (5.6)	77 (14.0)
More than 8 weeks	202 (56.4)	391 (71.0)
Never	120 (33.5)	5 (0.9)

### Pattern, prevalence and intensity of infection and co-infection

Across the two study sites, the combined prevalence of *P. falciparum* and *P. malariae* detected by microscopy was 4.2% (38/908) and 7.1% (26/364) by PCR. The combined methods yielded a total prevalence of 5.9% (54/908). A mixed infection with *P. falciparum* and *P. malariae* was seen in only one child. *P. faciparum* was detected in 29 children aged 5–14 years (29/630, 3.2%) and in 24 boys (24/569, 4.2%).

The overall prevalence of STH *spp* detected by microscopy in both sites was 1.4% (11/778) and 1.2% (9/767) by PCR. The combined methods showed a prevalence of 2.4% (19/787). *T. trichiura* was detected in six children (6/778, 0.8%), a mixed infection with *A. lumbricoides* and *T. trichiura* was detected in only one child. and *A. duodenale* was detected by PCR in one child. *T. trichiura* was slightly more common among children aged 5–14 years (10/577, 2.9%) than in 1–4 years old (2/208, 1.0%) and, in boys (9/515, 1.8%) than girls (3/270, 1.1%). A similar pattern of distribution was observed in the overall prevalence of any STH *spp*.

Fifty-one children had *S. haematobium* detected by the urine filtration test (51/857, 6%), 29 of whom had light and 22 had heavy infections. Eighteen children were detected to have *S. mansoni* by Kato-Katz method (18/857, 2.1%), all of whom had light infections. Utilizing PCR, 84 children had *S. haematobium* (84/868, 9.7%) and 41 children had a *S. mansoni* infection (41/868, 4.7%), giving an overall prevalence of 14.4%. The rapid POC-CCA test showed that 154 children had *Schistosoma spp* (154/874, 28.6%). *S. haematobium* infection was more common among 1–4 years old (37/254, 14.6%) and boys (69/558, 12.4%) than in 5–14 year old (65/618, 10.5%) or in girls (33/314, 10.5%). *S. mansoni* was also more common among 1–4 year olds (17/254, 6.7%) and in girls (24/314, 7.6%) than in 5–14 years (27/618, 4.4%) or boys (20/558, 3.6 %). The prevalence of mixed infection with *S. haematobium* and *S. mansoni* was 29% (253/874); with a higher prevalence observed in children aged 1–4 years (115/254, 45.3%) than in 5–14 year olds (138/618, 22.3 %) and in girls (111/314, 35.4%) compared with boys (141/558, 25.3%). More than half of the children had at least one intestinal protozoa (394/771, 51.1%). The prevalence of *Giardia intestinalis, Blastocytis hominis* and *Entamoeba coli* were 27.7, 22.2 and 18.9%, respectively. Intestinal protozoas were more common among children aged 5–14 years (279/569, 49%) than in 1–4 year olds (115/276, 56.9%) and in girls (138/338, 52.7 %) than in boys (253/507, 50.1%). The prevalence of polyparasitism *with P. falciparum*, STH, *S. haematobium* and *S. mansoni* among the children was 2.2% (20/910) while the prevalence of *P. falciparum-S. haematobium* co-infection was 1.1% (10/910), that of *P. falciparum-S. mansoni* 0.7% (6/910) and of *P. falciparum* with any intestinal protozoa 2.4% (22/910). Co-infection was higher among 5–14 year old children (17/629, 2.7%) than in 1–4 year olds (3/281, 1.1%) and in boys (13/567, 2.3%) than in girls (7/343, 2.1%) (Table 3).

**Table 3:** Prevalence of malaria, STH, schistosomiasis, intestinal protozoa and malaria-helminth co-infection by diagnostic methods, age group, and gender of study participants, Saraya and Diourbel, 2021.

	**Diagnostic method**				**Age group**			**Gender**	
		***N*** **(%, 95% CI)**				***N*** **(%, 95% CI)**			***N*** **(%, 95% CI)**	
**Malaria**	**Microscopy (*****n** =* **908)**		**PCR (*****n** =* **364)**	**Combined methods (*****n** =* **908)**	**1–4 years (*****n** =* **276)**		**5–14 years (*****n** =* **630)**	**Male (*****n** =* **569)**	**Female (*****n** =* **337)**
*P. falciparium*	32 (3.5 [2.45–4.97])		26 (7.1 [4.81–10.42])	32 (3.5 [2.45–4.97])	3 (1.1 [0.28–3.4])		29 (4.6 [3.14–6.58])	24 (4.2 [2.76–6.27])	8 (2.4 [1.10–4.79])
*P. malariae*	3 (0.3 [0.08–1.04])		0 (0)	3 (0.3 [0.08–1.04])	1 (0.4 [0.02–2.32])		2 (0.3 [0.05–1.26])	1 (0.2)	2 (0.6 [0.10–2.36])
*P.falciparum + P. malariae*	1 (0.1)		0 (0)	1 (0.1)	0 (0)		1 (0.2)	0 (0)	1 (0.3 [0.015–1.90])
***Any Plasmodium spp***	38 (4.2 [3.00–5.73])		26 (7.1 [4.81–10.42])	54 (5.9 [4.54–7.74])	7 (2.5 [1.11–5.38])		47 (7.5 [5.55–9.81])	38 (6.7 [4.80–9.09])	16 (4.8 [2.82–7.73])
**STH**	**Microscopy (*****n** =* **778)**		**PCR (*****n** =* **767)**	**Combined methods (*****n** =* **787)**	**1–4 years (*****n** =* **208)**		**5–14 years (*****n** =* **577)**	**Male (*****n** =* **515)**	**Female (*****n** =* **270)**
*T trichiura*	6 (0.8)		8 (1.0)	2(1.5)	2 (1.0)		10 (2.9)	9 (1.8)	3 (1.1)
*A. lumbricoides*	2 (0.3)		0 (0)	2 (0.3)	0 (0)		2 (0.6)	2 (0.4)	0 (0)
*A. lumbricoides + T. trichiura*	1 (0.1)		0 (0)	1 (0.1)	0 (0)		1 (0.3)	1 (0.2)	0 (0)
*Hymenolepis nana*	1 (0.1)		0 (0)	1 (0.1)	0 (0)		1 (0.3)	1 (0.2)	0 (0)
*Enterobius vermicularis*	1 (0.1)		0 (0)	1 (0.1)	0 (0)		1 (0.3)	1 (0.2)	0 (0)
*Ancylostoma duodenale*	0 (0)		1 (0.1)	1 (0.1)	0 (0)		1 (0.3)	0 (0)	1 (0.4)
***Any STH***	11 (1.4 [0.74–2.59])		9 (1.2 [0.57–2.30])	19 (2.4 [1.29–3.30])	2 (1.0 [0.17–3.80])		17 (2.9 [1.78–4.77])	15 (2.9 [1.70–4.87])	4 (1.5 [0.47–4.00])
**Schistosomiasis**	**Microscopy (*****n** =* **857)**		**PCR (*****n** =* **868)**	**POC–CCA (*****n** =* **539)**	**Combined methods (*****n** =* **874)**	**1–4 years (*****n** =* **254)**	**5–14 years (*****n** =* **618)**	**Male (*****n** =* **558)**	**Female (*****n** =* **314)**
*S. haematobium*	**Light**	29							
	**Moderate**	0							
	**Heavy**	22							
	**Total**	51 (6.0 [4.50–7.80])	84 (9.7 [7.83–11.89])		102 (11.7 [9.96–14.03])	37 (14.6 [10.59–19.65])	65 (10.5 [8.26–13.27])	69 (12.4 [9.81–15.45])	33 (10.5 [7.45–14.57])
*S. mansoni*	**Light**	18							
	**Moderate**	0							
	**Heavy**	0							
	**Total**	18 (2.1 [1.29–3.37])	41 (4.7 [3.45–6.41])		44 (5.0 [3.72–6.75])	17 (6.7 [4.07–10.69])	27 (4.4 [2.95–6.38])	20 (3.6 [2.26–5.58])	24 (7.6 [5.06–11.30])
**Any Schistosomiasis**	**Light**	47							
	**Moderate**	0							
	**Heavy**	22							
	**Total**	69 (8.1 [6.36–10.13])	125 (14.4 [6.36–10.13])	154 (28.6 [24.83–32.62])	253 (29.0 [25.98–32.10])	115 (45.3 [39.08–51.62])	138 (22.3 [19.15–25.86])	141 (25.3 [21.75–29.13])	111 (35.4 [30.12–40.95])
**Any helminths (STH** + **schistosomiasis)**	**Microscopy (*****n** =* **874)**		**PCR (*****n** =* **868)**	**All combined methods (*****n** =* **874)**	**1–4 years (*****n** =* **254)**		**5–14 years (*****n** =* **618)**	**Male (*****n** =* **558)**	**Female (*****n** =* **314)**
**Any helminths**	79 (9.0 [7.26–11.18])		126 (14.5 [12.27–17.08])	267 (30.6 [27.53–33.74])	117 (46.1 [39.84–52.40])		150 (24.3 [20.98–27.89])	152 (27.24 [23.63–31.17])	114 (36.3 [31.03–41.92])
**Intestinal protozoa**	**MIF (*****n** =* **771)**				**1–4 years (*****n** =* **276)**		**5–14 years (*****n** =* **569)**	**Male (*****n** =* **507)**	**Female (*****n** =* **338)**
*Giardia intestinalis*	214 (27.7)				75 (27.2)		139 (21.9)	130 (22.7)	83 (24.6)
*Entamoeba coli*	146 (18.9)				36 (13.0)		110 (17.4)	102 (17.8)	44 (13.0)
*Blastocystis hominis*	171 (22.2)				46 (16.7)		125 (19.7)	98 (17.1)	72 (21.3)
*Endolimax nana*	31 (4.0)				4 (1.5)		27 (4.3)	16 (2.8)	15 (4.4)
*Others e.g. Pseudolimax butschlii, Chilomastix mesnili*	34 (4.4)				9 (4.6)		25 (3.9)	18 (3.2)	16 (4.7)
**Any intestinal protozoa**	394 (51.1 [47.51–54.68])				115 (56.9 [49.78–63.80])		279 (49.0 [44.86–53.22])	254 (50.1 [45.66–54.53])	138 (52.7 [46.44–58.82])
**Co-infection**	***N** =* **910**				**1–4 years (*****n** =* **281)**		**5–14 years (*****n** =* **629)**	**Male (*****n** =* **567)**	**Female (*****n** =* **343)**
*P. falciparum* + STH + *S. haematobium + S. mansoni*	20 (2.2 [1.25–3.15])				3 (1.1 [−0.12–2.32])		17 (2.7 [1.43–3.97])	13 (2.3 [1.27–3.96])	7 (2.1 [0.52–3.48])
*P. falciparum* + *S. mansoni*	6 (0.7 [0.16–1.24])				1 (0.4 [−0.34–1.06])		5 (0.8 [1.0–1.48])	4 (0.7 [0.1–1.3])	2 (0.6 [−0.22–1.42])
*P. falciparum* + *S. haematobium*	10 (1.1 [0.42–1.78])				1 (0.4 [−0.34–1.06])		9 (1.4 [0.5–2.36])	8 (1.4 [0.4–2.37])	2 (0.6 [−0.22–1.42])
*P. falciparum* + any Schistosomes	19 (2.1 [1.17–3.03])				3 (1.1 [−0.12–2.32])		16 (2.5 [1.31–3.77])	12 (2.1 [0.9–3.28])	7 (2.1 [0.52–3.48])
*P. falciparum* + any intestinal protozoa	22 (2.4 [1.41–3.39])				0 (0)		22 (3.5 [2.06–4.92])	17 (3.0 [1.6–4.4])	5 (1.5 [0.19–2.62])

### Relationship between malaria parasitaemia and intensity of helminth infection

Table 4 summarizes the correlation of malaria parasite density with age group, gender and intensity of *S. haematobium* infection. The mean malaria parasite density was higher among participants aged 1–4 years than in children aged 5–14 years and in those who were girls and had light *S. haematobium* infection.

**Table 4:** Intensity of malaria parasitaemia (per μl of blood) in relation to age group, gender, and helminth infection status of study participants, Saraya and Diourbel, 2021.

	**Geometric mean of *P. falciparum* parasitaemia (95%CI)**	Mean difference^*^	***p*** **-value**
**Age group**			
1–4 years	3.10 (2.83–3.32)^†^		0.73
5–14 years	2.84 (2.72–2.98)	0.26	
**Gender**			
Female	2.83 (2.56–3.17)^†^		0.44
Male	2.88 (2.78–3.01)	0.05	
***S. haematobium***			
Light	2.79 (2.68–2.91)^†^		0.42
Heavy	3.14 (3.10–3.18)	0.35	

* Mean difference is the difference in means obtained by calculating the absolute difference between the mean values of the two groups.

† Reference variable.

### Risk factors for co-infection

Univariate analysis showed that participants aged 5–14 years, who were boys and lived in Saraya had a higher risk of malaria-helminth co-infection than their counterparts but this association did not a reach statistical significance. On the other hand, living in houses with spaces between the walls and roofs as well as frequent contacts with water during swimming were statistically significant risk factors for malaria-helminth co-infection. When adjusted for confounding factors, living in houses with spaces between the walls and roofs remained the only statistically significant risk factor for malaria-helminth co-infection (Table 5).

**Table 5:** Risk factors for malaria-helminth co-infections among study participants, Saraya and Diourbel combined, 2021.

**Characteristics**	**Unadjusted OR**		**Adjusted OR**	
	**(95%CI)**	***P*** **-value**	**(95%CI)**	***P*** **-value**
**Age groups**				
1–4 years	1.0 (ref)		1.0 (ref)	
5–14 years	2.50 (0.73–8.63)	0.14	3.37 (0.82–13.77)	0.09
**Gender**				
Female	1.0 (ref)		1.0 (ref)	
Male	1.10 (0.43–2.78)	0.84	1.44 (0.48–4.36)	0.51
**Site**				
Diourbel	1.0 (ref)		1.0 (ref)	
Saraya	1.97 (0.71–5.48)	0.19	1.27 (0.24–6.69)	0.77
**Space between the wall and roof**				
Don't Know	1.0 (ref)	**0.003**	1.0 (ref)	**0.006**
Yes	0.25 (0.07–0.97)		0.08 (0.01–0.49)	
No	0.09 (0.02–044)		0.31 (0.06–1.50)	
**Action when there was fever**				
No treatment given	1.0 (ref)	0.61	1 (ref)	0.09
Taken to health center	0.72 (0.20–2.57)		0.25 (0.05–1.24)	
Given herbal Preparations	2.00 (0.20–20.30)		2.76 (0.17–45.75)	
Treated at home with drugs bought from chemist	1.00 (0.16–6.13)		0.65 (0.06–6.39)	
Others	0.82 (0.08–8.08)		0.43 (0.03–5.70)	
**Contact with river e.g., swimming**				
Never	1.0 (ref)	**0.02**	1.0 (ref)	0.80
Everyday	4.65 (1.22–17.79)		1.33 (0.14–12.28)	
Approximately once in a week	1.33 (0.33–5.37)		0.45 (0.04–4.66)	
Rarely	1.39 (0.40–4.80)		0.59 (0.07–4.72)	
**Walking time to standing water source**				
< 5 min	1.0 (ref)	1.0 (ref)
5 to 10 min	0.32 (0.03–3.12)	0.33	0.50 (0.03–9.22)	0.64
10 to 14 min	0.87 (0.14–5.28)		1.14 (0.07–18.39)	
15 to 30 min	3.34 (0.87–12.84)		13.96 (1.18–165.06)	
>30 min	1.00 (0.25–4.05)		2.51 (0.23–26.85)	

P-values reached statistical significance because they were less than 0.05.

## Discussion

Comprehensive monitoring of control programmes targeting malaria and NTDs are crucial to assess the impact of the interventions, and align them toward achieving the new WHO road maps that now focus on elimination of these diseases by 2030. We conducted the studies in two epidemiological distinct yet similar settings in Senegal where malaria and NTD control programmes have been consistently implemented. Despite the rural and sub-urban locations of the two study sites, the socio-demographic features of the study participants were largely similar. However, disparities were observed in the fewer girls and relatively low number of children aged 1–4 years enrolled in Diourbel compared to Saraya where equal numbers of boys and girls were enrolled. One of the reasons for these differences is that the house-to-house recruitment approach adopted in the rural communities of Saraya was not logistically feasible in the sub-urban setting of Diourbel because of its cosmopolitan nature and relatively high population density. Hence, the recruitment of study participants was done through the Koranic school platform which enjoys a very high enrolment for pre-school and school-aged children in Senegal.

Also, despite the striking differences in the house structures in the two study sites, a similar proportion of children in Diourbel and Saraya lived in houses which had no net on the doors or windows. However, household ownership of, and sleeping under, a treated bed net was higher among Diourbel children than their counterparts in Saraya. A similar trend was observed in hand hygiene following defaecation where a relatively higher proportion of children in Diourbel practiced hand washing with soap and water.

Our findings showed that the prevalence of polyparasitism with *P. falciparum*, STH, *S. haematobium* and *S. mansoni* among the children was very low at 2.2%, despite the use of conventional and improved diagnostic methods such as PCR. Similar low trends were observed for co-infections with *P. falciparum-S. haematobium, P. falciparum-S. mansoni* and *P. falciparum* with any intestinal protozoa. These results contrast sharply with the findings of similar studies conducted in co-endemic communities in SSA (34–39). A plausible reason for the striking differences may be due to the fact that our studies were conducted in 2021 while previous studies reporting higher prevalence of *Plasmodium*-helminth co-infection were conducted between 2010 and 2015 when control programmes for malaria and NTDs were sub-optimal in many countries. The findings of a very low prevalence of the polyparasitism in our studies are likely to be a direct consequence of the impact of effective malaria and NTD control measures which included case-management with drug treatment, application of indoor residual spraying (IRS), distribution of insecticide-treated nets (ITN) and integrated vector management measures that were consistently implemented on a yearly basis in Senegal (20, 40). Similar findings have been reported in the neighboring Gambia where malaria (41, 42), and NTD (43) have also reached pre-elimination stages. However, a few recently conducted studies in other West African countries (44, 45) and in many other Sub-Saharan countries have shown that the prevalence of malaria is still very high, especially among pre-school and school aged children (46) Though not as high as the burden of malaria in these African countries, the epidemiological profile of STH and schistosomiasis are also higher than the findings of our studies. For example, a recently conducted study in a co-endemic community in Sierra Leone reported a malaria prevalence of about 30% among children aged 1–11 years and 12.5% for STH (44). Despite sharing similar epidemiological and environmental features, the findings from Sierra Leone differ remarkably from an overall prevalence of 3.5% obtained for *Plasmodium spp* across our two study sites. In the same vein, the overall prevalence of STH *spp* in our studies was about 1%, making our study areas a strong candidate for STH elimination as a public health problem (EPHP), as defined by the WHO NTD road map of attaining a prevalence of < 2% of moderate-to-heavy intensity infections (11). Although effective implementation of malaria and NTD control programmes have been widely cited as the major reason for the disparities in the burden of malaria and STH in our study areas and other African countries (9, 10, 12), more empirical studies are needed to unravel other contributing factors that may be responsible for the wide differences obtained in our study areas and other African countries where the burden of malaria-helminth co-infection is still high.

Unexpectedly, the prevalence of schistosomiasis in our studies did not follow a similar trend observed for malaria and STH. Given that mass drug administration of preventive chemotherapy for STH and schistosomiasis are implemented concurrently during national campaigns in Senegal, an overall prevalence of 28.6% by POC-CCA test suggested that schistosomiasis control is not as effective as for STH. Although, a prevalence of schistosome infection of 8% was obtained with the Kato-Katz method while PCR gave a prevalence of 14%, we did not observe in our study the limitations in diagnostic accuracy surrounding the use of POC-CCA rapid test in low endemic settings where low parasite burden is common (47). The Kato-Katz method is known to be less sensitive in low transmission settings, hence, its use yielded the lowest prevalence for detection of *Schistosomes* in our studies. The relatively high prevalence of schistosomiasis identified by PCR and POC-CCA tests in our studies might be explained from poor uptake of praziquantel among children. Due to its unpleasant taste, many children have been reported to spit or vomit praziquantel within few minutes of administration. A pediatric formulation of praziquantel has recently been developed and evaluated in clinical trials to overcome this limitation, but it will not be ready for deployment until 2024 (48).

Although, the prevalence of co-infection involving *P. falciparum* and intestinal protozoa was low in our studies, more than half of the children had at least one intestinal protozoa, with the prevalence of 27.7, 22.2 and 18.9% for *Giardia intestinalis, Blastocystis hominis* and *Entamoeba coli*, respectively. Despite Kato-Katz technique being the gold standard method recommended for detection of intestinal helminths, it did not identify any of these protozoas. This confirms the superiority of the MIF technique deployed in our studies, despite being a simpler and less expensive technique that performs competitively with Kato-Katz in both laboratory and field work on intestinal helminths, particularly in resource-limited settings (49). Although STH appears to be moving toward elimination stage in our study areas, the burden of multiple intestinal protozoa was still very high. Various factors in the biology of the intestinal protozoans, including the high excretion rate, low infectious dose, and the robustness of the cyst transmission stage, are particularly suited for transmission in our study sites. Most NTD control programmes deploy the WHO recommended albendazole or mebendazole as the preventive chemotherapy of choice for STH control, (50) which however is not effective against the intestinal protozoas. Given the need to tackle this silent burden of high intestinal protozoans by a cross-disciplinary and collaborative approach, an addition of a single-dose of tinidazole to the current NTD preventive chemotherapy may be justified, due to the challenges of poor compliance with multiple doses of metronidazole.

The distribution pattern of polyparasitism involving *P. falciparum*, STH, *S. haematobium* and *S. mansoni* in our studies showed a higher preponderance among 5–14 years old children and boys. The same patterns were also observed for co-infections with *P. falciparum*-STH, *P. falciparum- S. haematobium*, and *P. falciparum-S. mansoni*. These findings agree with the results of some other studies conducted in co-endemic settings in SSA (1, 51, 52), while a few other studies reported different distribution patterns (39, 53, 54). A main reason for the similarities of our findings with other studies is the high prevalence among male school-aged children which increased their risk to co-infection with *P. falciparum* and multiple helminths. Different socio-cultural practices which placed some activities such as fetching water on young girls, thereby exposing them frequently to contaminated water and increased their risk for the co-infection accounted for the different findings reported in other studies (39, 53, 54). Univariate analysis in our studies showed that living in houses with spaces in the roofs and walls as well as frequent contacts with contaminated water were the risk factors for malaria-helminth co-infection. However, when this was adjusted for confounding variables, living in houses with spaces in the roofs and walls remained the only predictor of malaria-helminth co-infection. These findings require further confirmation because the children might also be exposed to other environmental risk factors and/or risky sanitation and hygiene practices which were not investigated.

The major limitation of our studies was the different timing of the implementation of the two studies as the first study was conducted in Saraya at the beginning of malaria transmission season in June 2021, whilst the second study was conducted at the end of malaria transmission in Diourbel. These timings might have impacted on our findings but these times were selected to avoid the overlap with the implementation of SMC campaigns which run from June to September and the MDA campaigns for STH and schistosomiasis which are held annually around October in the study sites. To ensure that we obtained reliable estimates of the epidemiological profile of malaria-helminth co-infection, we deployed both conventional and improved diagnostic methods which strengthened our findings. We will also conduct further analysis using Luminex multiplex assays (55) to understand the exposure history of the children and the transmission dynamics of *Plasmodium* and helminth co-infection in our study areas.

## Conclusions

We found a very low prevalence of malaria-helminth co-infection in two diverse settings in Senegal, but the prevalence of mono-infection with schistosomiasis and intestinal protozoans was high among the pre-school and school-aged children. The incidence of co-infection was bound to be low because of the low prevalence of malaria. Low parasite loads could represent chronic parasite infections which may play a major role in multiple morbidities, the impact of which are often sub-clinical acting as reservoirs that make interruptions of cycles of infection transmission impossible. Therefore, more studies are needed to obtain reliable estimates of malaria-helminth co-morbidity in high and low transmission settings. This will provide country-specific evidence to guide the development of cross-cutting approaches that would address the critical gaps in implementation of strategies that may help achieve the WHO targets of eliminating malaria and NTD by 2030.

## Data availability statement

The original contributions presented in the study are included in the article/Supplementary material, further inquiries can be directed to the corresponding author.

## Ethics statement

The studies involving human participants were reviewed and approved by the London School of Hygiene and Tropical Medicine and the Comité National d'Ethique pour la Recherche en Santé (CNERS) in Senegal. Written informed consent to participate in this study was provided by the participants' legal guardian/next of kin.

## Author contributions

MOA conceptualized the study and developed the first draft of the manuscript. MOA, BG, JLAN, and DS developed the study protocol. MOA, JLAN, and DS supervised the data collection. IM, EBF, and IAM coordinated the field work. ACL, MPD, NAG, and AD performed the laboratory analysis. MAL analyzed the data. BG, JLAN, and DS reviewed the manuscript critically for important intellectual contents. All authors reviewed and approved the final draft of the manuscript.
