# Supplementary material for: Prevalence of malaria-helminth co-infections among children living in a setting of high coverage of standard interventions for malaria and helminths: Two population-based studies in Senegal
Source: Front Public Health. 2023 Mar 2;11:1087044. doi: 10.3389/fpubh.2023.1087044 (PMC10018210; doi:10.3389/fpubh.2023.1087044)
Supplement: Supplementary file 1 [file Data_Sheet_1.docx]

# Supplementary methods

1. Extraction of parasite DNA

The malaria parasite genomic DNA was extracted from two 3 mm punches of dried blood blots using Saponin-Chelex extraction. [1] Briefly, the blood-stained filter paper discs for each sample were incubated in 1.5 ml containing 0.8 ml of 0.5% saponin/PBS solution overnight at room temperature on a shaking incubator. After the overnight incubation, the supernatant was discarded and the samples washed twice with 0.8 ml PBS, followed by a high-speed centrifugation at 10,000×g. Finally, 75 μl of 20% Chelex (Sigma-Aldrich, USA) in 150 μl DNase/RNase free water was added to the washed samples and incubated at 95 °C for 8 minutes to extract DNA from the samples. After a final high-speed centrifugation, the supernatant containing the DNA was stored at −20 °C until used for the genotyping amplification reactions.

1.1: *Plasmodium falciparum* prevalence by real-time PCR

The presence of *P. falciparum* was determined by real-time PCR with species-specific primers based on 18s rRNA gene, as described by Rougemont et al. [2] The real-time reaction mixture was prepared using 5µl of using Thermo Scientific luminaris Probe qPCR Master Mix (2X), 0.3 µl of each primer (10 µM), 0.2 µl of probe (10 µM), 4.2µl of nuclease free water and 2.5 µl of the extracted template DNA giving a total volume of 12.5 µl. The primers and probes used are described in Table S1.

**Table S1: Primers and probes for *Plasmodium* real-time PCR analysis**

| Plasmo2-R primera | AAC CCA AAG ACT TTG ATT TCT CAT AA |
| --- | --- |
| Fal-F primer | CCG ACT AGG TGT TGG ATG AAA GTG TTA A |
| Falc-probeb | Quasar 670-AGC AAT CTA AAA GTC ACC TCG AAA GAT GAC T-BHQ-2 |

1.2: Cycling condition

The real-time PCR assay was performed on a CFX96^TM^ machine (BIO-RAD, Life Science, Marnes-la-Coquette, France). The cycling was done as follows: 2 minutes UDG pre-treatment at 50°C, 10 minutes initial denaturation at 95°C followed by 40 cycles of 15 seconds denaturation at 95°C and 60 seconds hybridisation at 60°C. *P. falciparum* 3D7 collected from parasite culture at San Francisco University (USA), was used as DNA positive control while nuclease free water was used as a negative control.

2. Stool and urine DNA extraction

DNA was extracted from urine and stool samples using Kit ZymoBIOMICS ^TM^ DNA Miniprep Kit (Zymo Research, California, USA). The extraction, purification and elution of the DNA were done following the manufacturer’s recommendations. For stool samples, mechanical lysing using a fast-prep machine was also done.

2.1: PCR assays

**Urines:** The real time PCR assay was done targeting the *Schistosoma haematobium* Dra1 gene, as previously described. [3] Primers and probes sequences were as follows Sh-FW: 5′-GATCTCACCTATCAGACGAAAC-3′; Sh-RV: 5′-TCACAACGATACGACCAAC-3′; Sh-probe: 5’-FAM-TGTTGGTGGAAGTGCCTGTTTCGCAA-TAMRA-3’. The real-time reaction mixture was prepared using 5µl of Thermo Scientific luminaris Probe qPCR Master Mix (2X), 0.3 µl of each primer (10 µM), 0.2 µl of probe (10 µM), 4.2µl of nuclease free water and 2.5 µl of the extracted template DNA giving a total volume of 12.5 µl.

**Stool samples:** Primers and probes are illustrated in Table S2.

**Table S2: Primers and probes used for stool PCR analysis**

| **Species** | **Primer and Probe name** | **Sequence 5' - 3'** |
| --- | --- | --- |
| *Ascaris lumbricoides* | Alum96F: | GTAATAGCAGTCGGCGGTTTCTT |
| *Ascaris lumbricoides* | Alum183R: | GCCCAACATGCCACCTATTC |
| *Ascaris lumbricoides* | Alum124T: | FAM-TTGGCGGACAATTGCATGCGAT-TAMRA |
| *Trichuris trichiura* | TrichF: | TTGAAACGACTTGCTCATCAACTT |
| *Trichuris trichiura* | TrichR: | CTGATTCTCCGTTAACCGTTGTC |
| *Trichuris trichiura* | TrichP: | ROX-CGATGGTACGCTACGTGCTTACCATGG- |
| *Strongyloides* | Stro-1530F: | GAATTCCAAGTAAACGTAAGTCATTAGC |
| *Strongyloides* | Stro-1630R: | TGCCTCTGGATATTGCTCAGTTC |
| *Strongyloides* | Stro-1586T: | VIC-ACACACCGGCCGTCGCTGC- |
| *A. duodenale* | Ad125F: | GAATGACAGCAAACTCGTTGTTG |
| *A. duodenale* | Ad195R: | ATACTAGCCACTGCCGAAACGT |
| *A. duodenale* | Ad155-XS: | FAM-ATCGTTTACCGACTTTAG-MGB |
| *N. americanus* | Na58F: | CTGTTTGTCGAACGGTACTTGC |
| *N. americanus* | Na158R: | ATAACAGCGTGCACATGTTGC |
| *N. americanus* | Na81Tmgb: | ROX-CTGTACTACGCATTGTATAC-MGB |
| *S. mansoni* | SRA1 | CCACGCTCTCGCAAATAATCT |
| *S. mansoni* | SRS2 | CAACCGTTCTATGAAAATCGTTGT |
| *S. mansoni* | SRP | VIC-TCCGAAACCACTGGACGGATTTTTATGAT- |
| Toute bacterie | Toutbact_16S_F: | AGAGTTTGATCMTGGCTCAG |
| Toute bacterie | Toutbact_16S_R: | TTACCGCGGCKGCTGGCAC |
| Toute bacterie | BACT338K: | CY5-CCAKACTCCTACGGGAGGCAGCAG |

A multiplex PCR was carried out on the stools samples. Two groups were formed according to the dyes. The first group was *Ascaris lumbricoides, Trichuris trichiura, Schistosoma mansoni* and “Toute bacterie” that is, extraction control. The second group consisted of *Strongyloides stercoralis, Ancylostoma duodenale* and *Necator americanus.*

**Group 1:** The real-time reaction mixture was prepared using 5µl Thermo Scientific luminaris Probe qPCR Master Mix (2x), 0.3 µl of each primer (10 µM) for each species, 0.2 µl of each probe (10 µM) for each species, 1.8 µl of nuclease free water and 2.5 µl of the extracted template DNA giving a total volume of 12.5 µl. The target gene for *Ascaris lumbricoides* was ITS1, for *Trichuris trichiura* , it was 18S and for *Schistosoma mansoni,* it was tandem repeat units.

**Group 2:** The reaction mixture for the PCR was prepared using 5µl of Thermo Scientific luminaris Probe qPCR Master Mix (2X), 0.3 µl of each primer (10 µM) for each species, 0.2 µl of each probe (10 µM) for each species, 4.2 µl of nuclease free water and 2.5 µl of the extracted template DNA giving a total volume of 12.5 µl. The target gene for *Strongyloides stercolaris* was 18S and ITS2 for *Ancylostoma duodenale* and *Necator americanus.*

2.2: Cycling condition for urine and stool samples

The real-time PCR assay was performed on a CFX96^TM^ machine (BIO-RAD, Life Science, Marnes-la-Coquette, France). The cycling was done as follows: 2 minute UDG pre-treatment at 50°C, 10 min initial denaturation at 95°C followed by 40 cycles of 15 seconds denaturation at 95°C and 60 seconds hybridisation at 60°C. Positive DNA control for all species used in this study were obtained from previous positive cases. Nuclease free water was used as a negative control.

2.3: Interpretation of results

Samples were considered as positive if the cycle threshold (Ct) was below 35. For Cts between 35 and 38, the amount of DNA was doubled. In this case, the curve shift by 3 CT (±1) led to the sample being considered positive.

# References

1. Baidjoe A, Stone W, Ploemen I, Shagari S, Grignard L, Osoti V, et al. Combined DNA extraction and antibody elution from filter papers for the assessment of malaria transmission intensity in epidemiological studies. Malar J. 2013; 12:272.

2. Rougemont M, Van Saanen M, Sahli R, Hinrikson HP, Bille J, Jaton K. Detection of four Plasmodium species in blood from humans by 18S rRNA gene subunit-based and species-specific real-time PCR assays. Journal of clinical microbiology. 2004;42(12):5636-43. Epub 2004/12/08. doi: 10.1128/jcm.42.12.5636-5643.2004. PubMed PMID: 15583293; PubMed Central PMCID: PMCPMC535226.

3. Ibironke OA, Phillips AE, Garba A, Lamine SM, Shiff C. Diagnosis of Schistosoma haematobium by detection of specific DNA fragments from filtered urine samples. The American journal of tropical medicine and hygiene. 2011;84(6):998-1001. Epub 2011/06/03. doi: 10.4269/ajtmh.2011.10-0691. PubMed PMID: 21633040; PubMed Central PMCID: PMCPMC3110375.
